# Causal Overgeneralization of COVID-19 Vaccine Adverse Events Undermines Scientific Integrity: A Technical Report

**DOI:** 10.7759/cureus.82121

**Published:** 2025-04-11

**Authors:** Debashis Priyadarshan Sahoo

**Affiliations:** 1 Internal Medicine, All India Institute of Medical Sciences, Guwahati, Guwahati, IND

**Keywords:** adverse event, covid 19, covid-19 vaccine, rare side effect, vaccine science and policy

## Abstract

Public discourse surrounding COVID-19 vaccines has often been marred by the overgeneralization of adverse events, where any health issue following vaccination is presumed to be vaccine-induced. This misattribution undermines pharmacovigilance efforts, making it harder to identify true safety signals, while fueling misinformation. Given the large-scale, global vaccination effort, coincidental health events are inevitable and must be distinguished from genuine adverse reactions using robust epidemiological and mechanistic assessments. This editorial highlights the importance of statistical rigor in vaccine safety evaluations, emphasizing the need for objective, evidence-based analysis to maintain scientific integrity and public trust.

## Editorial

The pervasive tendency to attribute all post-vaccination health events, from nonspecific symptoms to acute cardiovascular syndromes, to COVID-19 vaccines reflects a critical lapse in scientific reasoning. With over 13.6 billion doses administered globally by mid-2024, the vaccinated population’s scale ensures probabilistic overlap with unrelated pathologies [1]. This confounds signal detection in pharmacovigilance systems and necessitates rigorous epidemiological and mechanistic analysis to distinguish vaccine-related effects from background noise.

Phase III randomized controlled trials (RCTs) of mRNA vaccines established a favorable safety profile, with serious adverse events not exceeding placebo rates beyond transient reactogenicity [1,2]. Post-marketing surveillance has since identified rare but significant risks: myocarditis/pericarditis at 3.2 per 100,000 doses in males aged 12-29, and thrombosis with thrombocytopenia syndrome (TTS) following adenoviral vaccines at 3.5 per 1,000,000 doses [3,4]. Mechanistically, mRNA vaccine-induced myocarditis may stem from transient immune activation via Toll-like receptor pathways, while TTS involves anti-platelet factor 4 antibody formation [4]. These risks, however, are orders of magnitude lower than sequelae of COVID-19: myocardial injury in 20%-30% of hospitalized cases, and thromboembolism at 150-200 per 100,000 [3].

Causal inference requires more than temporality. Baseline incidence rates, e.g., ischemic stroke (100-300/100,000/year) or sudden cardiac death (50-100/100,000/year), predict millions of coincidental events post-vaccination in a cohort of billions. Self-controlled case series (SCCS) and disproportionality analyses in systems like the Vaccine Adverse Event Reporting System (VAERS) and EudraVigilance are essential to isolate signals. A recent meta-analysis of mRNA vaccine safety reported no excess all-cause mortality, while fertility studies found no impact on fecundability, debunking widespread anecdotal claims [3].

This overgeneralization carries scientific and societal costs. It obscures genuine signals in adverse event databases, diverts resources from multifactorial diseases such as long COVID, and erodes trust in vaccines with proven efficacy [1-3]. Historical precedents, such as the 1976 H1N1 vaccine’s Guillain-Barré syndrome cluster, underscore the need for vigilance, but hypotheses must be tested via cohort studies, genomic profiling, and proteomic assays - not assumed via post hoc ergo propter hoc fallacies [5].

Regulatory transparency and independent research remain paramount. Studies of mRNA vaccine pharmacokinetics and adenoviral vector immunogenicity provide mechanistic clarity, yet gaps persist in long-term rare event detection [4]. Misattribution, however, bypasses this process, risking regression to pre-scientific heuristics. The vaccines’ impact - averting an estimated 20 million deaths by 2022 - demands we contextualize risks against benefits with precision [3].

We urge the scientific community to ignore causal overgeneralization, prioritizing specific, evidence-based investigations of adverse events. Statistical rigor and biological plausibility must guide discourse - not speculation - to safeguard both public health and the integrity of medical science.
